# Metal Nanomaterials and Hydrolytic Enzyme-Based Formulations for Improved Antifungal Activity

**DOI:** 10.3390/ijms241411359

**Published:** 2023-07-12

**Authors:** Ilya Lyagin, Aysel Aslanli, Maksim Domnin, Nikolay Stepanov, Olga Senko, Olga Maslova, Elena Efremenko

**Affiliations:** Faculty of Chemistry, Lomonosov Moscow State University, Lenin Hills 1/3, 119991 Moscow, Russia; lyagin@mail.ru (I.L.); ayselaslanli@mail.ru (A.A.); domninmaxchem@gmail.com (M.D.); na.stepanov@gmail.com (N.S.); senkoov@gmail.com (O.S.); olga.maslova.rabota@gmail.com (O.M.)

**Keywords:** green synthesis, MOFs, amyloid proteins, prionase, mycotoxins, growth inhibition, quorum sensing, quorum quenching, lactamases, biofilms, chitinases, lactonases, His_6_-OPH

## Abstract

Active research of metal-containing compounds and enzymes as effective antifungal agents is currently being conducted due to the growing antifungal resistance problem. Metals are attracting special attention due to the wide variety of ligands that can be used for them, including chemically synthesized and naturally obtained variants as a result of the so-called “green synthesis”. The main mechanism of the antifungal action of metals is the triggering of the generation and accumulation of reactive oxygen species (ROS). Further action of ROS on various biomolecules is nonspecific. Various hydrolytic enzymes (glucanases and proteases), in turn, exhibit antifungal properties by affecting the structural elements of fungal cells (cell walls, membranes), fungal quorum sensing molecules, fungal own protective agents (mycotoxins and antibiotics), and proteins responsible for the adhesion and formation of stable, highly concentrated populations in the form of biofilms. A wide substrate range of enzymes allows the use of various mechanisms of their antifungal actions. In this review, we discuss the prospects of combining two different types of antifungal agents (metals and enzymes) against mycelial fungi and yeast cells. Special attention is paid to the possible influence of metals on the activity of the enzymes and the possible effects of proteins on the antifungal activity of metal-containing compounds.

## 1. Introduction

The accumulation of information about the role that microscopic fungi can play in the development of a number of negative processes affecting human health [1,2,3] has led to increasing interest in antifungals that can control and reduce the growth, as well as the metabolic activity, of these biological objects, especially those associated with pathogens [4]. The seriousness of these tasks is increasing due to the fact that in some cases, fungal cells may develop resistance to the chemical formulations used against them [5,6,7].

A number of current scientific studies are related to the development of effective antifungals [8]. Among the new trends in the development of effective antifungals, the prospects of a possible combination of various chemical compounds [7] with different mechanisms of action on fungal cells are being considered. This approach can enable researchers to overcome the development of adaptive processes in fungi and, possibly, reduce the doses of the substances used, increasing the effectiveness of their action in such combinations. When implementing such a combined approach to suppressing the growth and metabolic activity of fungi, the main question arises about what is better to combine with what, and what may be unpromising. One of the possible answers to this question is based on the use of metal nanomaterials such as metal-nanoparticles, metal-organic frameworks, etc., to which no resistance is formed by most microorganisms since the mechanism of suppression of biological processes is primarily associated with the generation of reactive oxygen species (ROS) in the cells. Metals such as Ag, Cu, Fe, Zn, Se, Ni, Au, Zr, Ce, Ti, and Pd have been studied in regard to compounds possessing antifungal activity [9,10,11,12]. At the same time, current scientific research on the antifungal properties of metals is mainly focused on the study of Ag and Au nanoparticles (NPs) [10,11,12,13,14,15] since the antimicrobial effectiveness of their action has been known for a long time.

Among the various organic synthetic ligands for the metals used in research in this direction, the so-called “green synthesized” metal-containing NPs should be noted. These “green synthesized” metal-containing NPs are formed inside the cells of microorganisms (bacteria, fungus, yeast) in vivo or using plant extracts, polysaccharides of phototrophic microorganisms, and extracellular enzymes of mycelial fungi [10,14,15,16,17]. “Green synthesis” is an environmentally friendly synthesis technique that avoids the formation of undesired by-products and costs less. Moreover, it was found that “green synthesis” makes it possible to obtain NPs with identical antifungal properties compared to similar chemically synthesized metal-containing compounds that are, in some cases, superior to them [17].

It is known that the combination of metal NPs with known chemical fungicides makes it possible to reduce the minimum inhibitory concentration (MIC) of the latter by more than eight times [17]. However, despite this, in this review, we decided to consider the possibility of combining metal-containing compounds with biological molecules having catalytic properties, in particular, with various enzymes exhibiting antifungal activity instead of chemically synthesized fungicides. It has been previously shown that the efficiency of the use of metal NPs can be increased by combining them with cyclic peptides that exhibit antifungal properties [18]. Unlike peptides that exhibit antimicrobial activity, the enzymes have catalytic activity [19], which allows them not just to trigger destructive processes against fungi but to repeatedly participate in these acts of biocatalysis, deepening antifungal processes. In addition, a wide substrate range of action of the enzymes themselves allows us to consider the possibility of not only their destructive activity against fungal cells but also against the most important fungal molecules involved in the formation of their quorum sensing (QS) and adhesion [20] and molecules that ensure their own safety (antibiotics [21] and mycotoxins [22]).

In this regard, both from a scientific and practical point of view, it was interesting to analyze the current scientific background in the field of creating possible combinations of metal-containing compounds with enzymes possessing antifungal activity, as well as to evaluate the potential available in this area that can be used in practice. This was the purpose of this review. In order to better understand the existing prospects and limitations for the development of these combinations, in this review, we first paid special attention to recent information about metal nanomaterials and enzymes, on which, in recent years, the researchers concentrated when studying their antifungal characteristics. At the same time, in the case of enzymes, special attention was paid to the effect of different metals on their established antifungal activity, if such information was present in the analyzed publications.

## 2. Antifungal Agents Based on Metal Nanoparticles, Metal–Organic Frameworks and Their Composites

**Figure 1:**
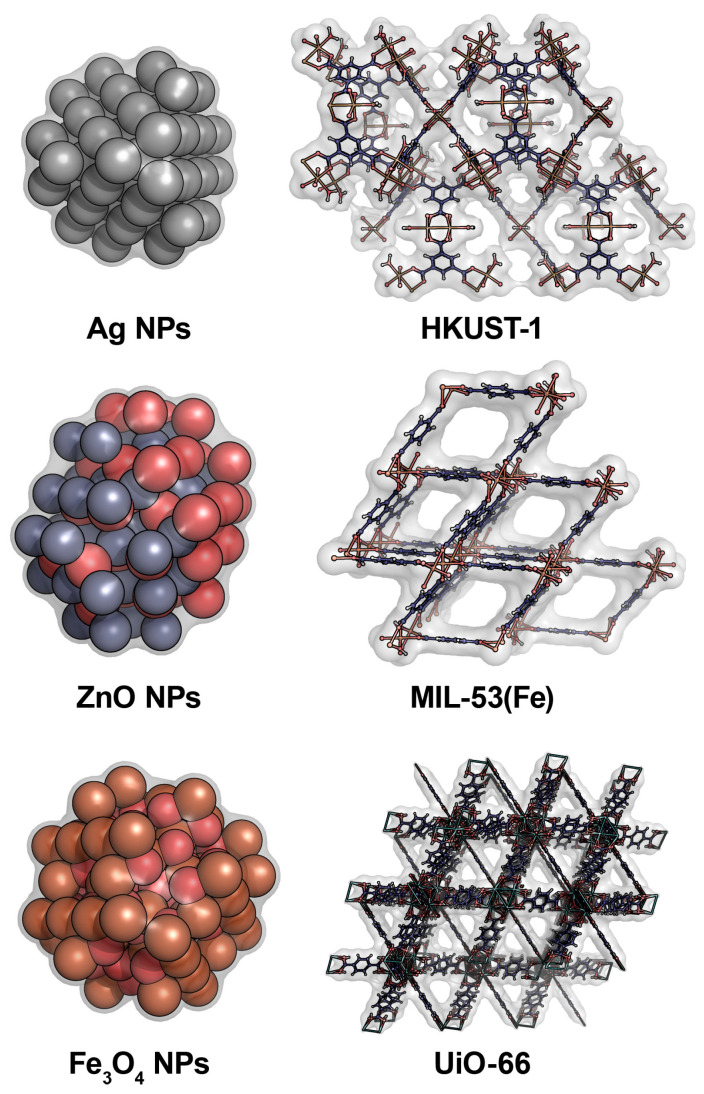
Some representative metal NPs and MOFs with antifungal activities. Crystal structures of Ag (1741252), ZnO (13950), Fe_3_O_4_ (1612598), HKUST-1 (2091261), MIL-53-Fe (2088536), and UiO-66 (2054314) were obtained from CCDC, then expanded in Mercury (v.4.2.0, CCDC, Cambridge, UK) and visualized in PyMOL (v.1.7.6, Schrödinger Inc., New York, NY, USA). Water-accessible molecular surface is indicated by light grey while atoms are colored by element: Ag–grey, Zn–slate, O–red, Fe–orange, C–deep blue, H–white, Zr–cyan.

Multiple antifungal agents have been developed to date on the basis of metal nanoparticles (NPs) and/or metal–organic frameworks (MOFs) (Table 1, [11,12,23,24,25,26,27,28,29,30,31,32,33,34,35,36,37], Figure 1).

### 2.1. Metal NPs

Relatively easily accessible metals, like Zn [24,26,27,28,29] and Fe [30,31] and their oxides, are dominantly used to produce antifungal NPs (Figure 1). Such metals are also a little bit more tolerable to non-target organisms. The most common mechanism of antifungal action is considered to be the generation of reactive oxygen species (ROS) oxidizing and thus damaging multiple targets within fungal cells [38]. However, the multitarget action of metal NPs is still possible.

For example, actively studied Ag NPs were found to inhibit the expression of genes of proteins involved in the biosynthesis of fatty acids and lipids, which are components of membranes [39]. It is believed that a more potent manifestation of antifungal properties is stimulated by the ionization of metals, for example, under the action of oxygen dissolved in the medium. Silver ions are able to bind almost irreversibly to thiol-containing compounds, e.g., cysteine, glutathione, coenzyme A, etc., which are vital components of fungal cells. As a result, many metabolic pathways and cellular structures can be affected. The advantage of metal NPs is the possibility of their enhanced adherence to the cell surface. In this way, it is possible to increase their local concentrations, as well as facilitate the intracellular penetration of NPs and metal ions.

It should be noted that some fungi, for example, representatives of the genera *Alternaria* and *Aspergillus*, have a tolerance to the toxic effect of metals, particularly Cu^2+^ and Pb^2+^ ions [40], whereas the high enough toxicity of the same metals may be obvious for many ecosystem participants, including humans. The strategies of fungal tolerance to metals are numerous, and the formation of complexes with various biomolecules and the active outflow or excretion of metals from the cells could be highlighted [40]. In this regard, it is interesting to search for those metal-containing formulations that also target such resistance pathways.

Special efforts are being made to create nanomaterials with intrinsic enzyme-like characteristics, which are called “nanozymes” [41]. Nanozymes successfully combine the unique properties of nanomaterials and mimic the catalytic functions of enzymes [41]. Known nanozymes with antifungal properties can mimic the action of peroxidases, catalases, and superoxide dismutases [11,12,36,37]. Such nanozymes can be synthesized on the basis of Ce [11], Ag, Fe [36,42], Pt, Pd, Cu, Ti [12], and some other metals [41]. Combined nanozymes are expected to have higher efficiency and an expanded spectrum of antimicrobial action, as in the case of Fe_3_O_4_ [36] and TiO_2_ [12].

Regarding the future prospects for the development of new nanozymes, another potential target for their action is the fungal cell wall. The cell wall of the most fungi is mainly composed of chitin and β-1,3-glucan, both of which can be hydrolyzed (see below). In this regard, novel antifungal nanozymes may have not only oxidoreductase but also hydrolase-like activity [41].

Intriguingly, such metal-containing catalysts have been developed for use in another field, particularly for the destruction of microplastics [43]. It would be extremely interesting to test metal-containing nanozymes, already developed for other purposes, as antifungal agents.

### 2.2. MOFs

Multiple methods and metals are used for the synthesis of MOFs with antifungal activity [11,32,33,34,35,44]. Ce [11] and Cu [32,34,44] are the most often applied metals. Meanwhile, a wide range of organic linkers can be implemented, for example, 4,4′,4″-nitrilotribenzoic acid [11], 1,4-benzene dicarboxylate [35], 1,3,5-benzene tricarboxylate [32,44], and glutarate together with 1,2-bis(4-pyridyl)ethane, 1,2-bis(4-pyridyl)ethylene, or 1,3-bis(4-pyridyl)propane [34], and others [45].

As in the case of metal NPs, MOF-based antifungals have different mechanisms of action. For example, the reduction of metal ions in the composition of MOFs can generate superoxide radicals and other ROS, followed by the degradation of biomolecules and the inhibition of cell growth [32]. Under certain conditions, metal ions are released from MOFs into the environment with fungi and possess a toxic effect on them [33]. However, not all MOFs have antifungal properties. Apparently, the type of metal in the MOFs’ composition has a key role in this case.

Moreover, MOF particles can be synthesized in nanosized range while giving them antifungal activity (Figure 1). Thus, NPs of UiO-66 (zirconium-based MOFs, 68.4 ± 8.5 nm) when injected into a medium with white rot fungus *Phanerochaete chrysosporium* showed a weak effect on cells and did not penetrate them but slightly damaged the cell wall and inhibited the activity of the laccase secreted by them [46].

### 2.3. Green Synthesis of NPs and Its Influence on Mechanisms of Their Antifungal Action

Recently, the green synthesis of nanomaterials has been proposed as an alternative to conventional chemical synthesis. By design, the green synthesis and biosynthesis of NPs can result in the inclusion and/or adsorption of multiple organic compounds present in the culture broth and cells, including proteins, among others. Usually, it is hard to distinguish the contribution of such protein(s) to the antifungal activity of the end product. Thus, many authors did not try to. However, to date, many such (nano)formulations have been developed using microalgal [10,47], bacterial [48,49], fungal [50], plant [51,52], and other isolates. A slightly more accurate method applies, at least, partially purified proteins [53,54] and pure polypeptides [55] for the green synthesis of NPs.

Thus, different metal NPs can be synthesized via the green route (Table 2 [10,47,48,49,50,51,52,53,54,55]). For example, the application of Ag led to the profound antibacterial activity of obtained formulations [48,52], thus giving additional functionality and opening an opportunity to treat multi-microbial associations. Antioxidant activity is another known mode of action and can be intrinsic to formulations due to the metals/methods used [51,52] or can be provided by additional components, e.g., phenol-containing antioxidants [52,53].

Considering the mechanism of antifungal action, the most often used and thus deeply investigated yeasts belong to the genera *Candida* (Table 1 and Table 2). They are commensal microorganisms in humans, but sometimes, a few of them transform into pathogens [56]. As a consequence, they are evasive to most immune responses. Moreover, they possess the ability to form biofilms that are more resistant to antifungal treatment(s) and the ability to ultimately transform into (pseudo)hyphal morphology [57]. Thus, the best efforts are being directed toward the dispersion of biofilms and, at most, toward the inhibition of yeast to hyphal transition [50,51].

Mechanistically, both formulations seem to affect the structural integrity and/or permeability of cell membranes and stimulate an increase in ROS, followed by cumulative damage. Under similar circumstances, farnesol is known to downregulate multiple genes, including those responsible for biofilm formation and preservation [56]. To date, yet another interesting option not considered is the direct interaction of metal-containing nanomaterials with adhesins belonging to the family of agglutinin-like sequence (ALS) proteins and forming nanodomains on cell surfaces. Indeed, ALSs are long-known multipurpose utilities that participate in biofilm formation/adhesion and are also capable of binding with ferritin [58] and even macroscopic metal surfaces [59]. Interestingly, interaction with metal surfaces similarly results in ROS generation and the modulation of several genes, particularly issued in this work, responsible for intracellular metal homeostasis.

Filamentation is the next step of yeast biofilm maturation after initial cell attachment. It, at least partially, depends on cascades with ALSs, and thus, disturbing their regulation could lead to the inhibition of (pseudo)hyphal formation. Strong evidence of such inhibition is shown with Ag NPs [50] and Fe_3_O_4_ NPs [51]. Noteworthily, medium-chain fatty acids (e.g., heptanoic and nonanoic acids) and, to a lesser extent, farnesol molecules not only downregulate genes responsible for hyphal formation but also upregulate genes responsible for yeast-cell morphologic form [60].

Thus, a cumulative effect can occur due to proteinaceous, fatty acids, and other biologically active compounds forming a corona of green synthesized nanomaterials. It should be noted that a combination of formulation with conventional antifungals (e.g., fluconazole) acting on another biochemical target(s) within yeast resulted in a synergetic effect and also improved efficiency [50].

Many filamentous fungi have a spore stage in which germination can be triggered by different biotic and abiotic factors [61]. This is a hard target to influence, but it is still possible to affect it using, for example, nanomaterials [39]. Thus, there are several pathways, at least, to prevent the activation of the process and, at most, to irreversibly disable it, for example, while degrading the spores. The latter case is rather limited now due to (bio)compatibility issues and is a matter of future research. Germination is initiated when outer and/or inner receptors are activated by the factor or when autoinhibition is eliminated by it.

Due to their small size, NPs can be adsorbed on the surface of spores and visualized there [49]. After that, physical damage and/or hindrances to the penetration of activating factors, as well as chemical modification(s) of the spore envelope, outer receptors, and transport channels are possible. The internal resources of spores are limited, and they cannot sustain a constant regeneration process. As a result, many of them may lose the ability to germinate properly, while the remaining ones may be severely depleted, which, for example, appeared in the production of decreased amounts of extracellular enzymes [49]. Moreover, additional functionalities of antifungal formulations, e.g., ROS generation, can be very useful to improve the rate and efficiency of the process of spore inactivation [55]. It should be noted here that measured values of leaked proteins and nucleic acids from the spores [49] seem to be unreliable and should be verified.

A similar mechanism of action of nanomaterial formulations is even more profound for vegetative filamentous fungi [55]. Structurally mycelium is less durable than spores, while the visualization and/or measurement of inflicted damages is easier. Noteworthily, proteinaceous corona improves anti-sporulation, anti-germination, and other effects of the antifungal formulations in multiple times.

Another important point about the possible toxicity of prepared formulations to humans and other non-target organisms is worth mentioning. Some toxicity is shown for Ag NPs towards mouse fibroblasts, human lung carcinoma epithelial cells, and human red blood cells [50] and for Ti/Ag NPs towards human skin fibroblasts [49]. The formulation of CuO with poly-ε-lysine [55] has no influence on seed germination.

Prepared antifungal formulations were tested *in planta* in the soluble form for prophylaxis and therapy [55], in the coating films for fruits [53], in the nanofibrillar package material [54], etc. However, some reports [53,58] appeared to be rather preliminary without special contamination by fungal cells. Applications in prophylaxis and therapy modes led to very similar antifungal activity [55], but the removal of fungi was incomplete at the applied doses.

A few words should be mentioned about the determination of antifungal activity in different works. Many authors still use the disk diffusion test (Table 2), which is useful for medical diagnostics but insufficient for the research field. (Micro)dilution tests seem to be much more preferable, especially in the case of non-filamentous fungi. Tests of spore germination and/or mycelium growth were applied for filamentous fungi. Nevertheless, all these results did not allow for distinguishing between the true killing of cells to death and cells in the stasis state. Modern tests determining the real cellular state, like the live/dead assay, ATP test [62], etc., should be widely implemented.

In regard to possible perspectives of future research, the specific modification of metal NPs for the targeted delivery of antifungal formulation, as it was realized in the case of organic liposomes [63], seems to be a very interesting direction. It could reduce the off-target toxicity discussed earlier and improve the efficiency of antifungal nanoformulation(s).

## 3. Enzymes as Antifungal Agents

### 3.1. Antifungal Enzymes Using Cell Structural Components of Fungi as Substrates

**Figure 2:**
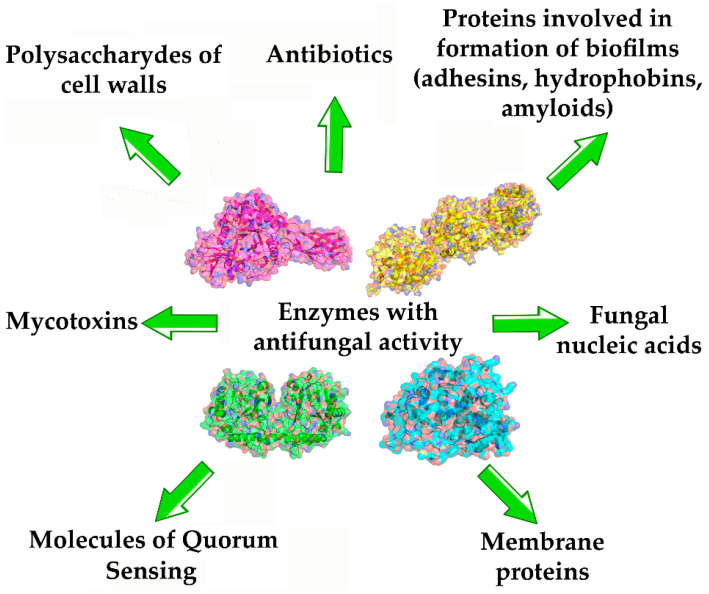
Enzymes (pink—chitinase (PDB ID: 1edq); yellow—keratinase (PDB ID: 5wsl); green—lactonase AidC (PDB ID: 4zo2); blue—peroxidase (PDB ID: 1mnp) with antifungal activities due to their catalytic action on different targets as substrates.

Discussing the possibilities and prospects for the use of various enzymes in the composition of antifungals, it should be noted that their diversity is determined by the spectrum of targets on which these enzymes can have notable effect, leading to a halt in the fungal growth, disruption of metabolism and death of fungi. Such targets for enzymatic action may include: structural elements of fungal cells (cell walls, membranes) [64], nucleic acids [65], fungal Quorum Sensing molecules (QSMs) regulating fungal resistance to various negative factors and protect them (mycotoxins, antibiotics) [66]), peptides and proteins involved in the formation of stable forms such as biofilms (adhesives, hydrophobins) (Figure 2).

Enzymes are interesting as antifungal agents because they are proteins present in various natural sources (plants, microorganisms, animal tissues) (Table 3 [67,68,69,70,71,72,73,74,75,76,77,78,79,80,81,82,83,84,85,86,87,88,89,90,91,92,93,94,95,96,97,98,99]), which are designed to protect living objects from the effects of fungi. The recombinant forms of necessary enzymes can be produced in various host cells.

The analysis of enzymes exhibiting a variety of antifungal activities indicated that most of them were hydrolases acting on polysaccharides present in the structure of the fungal cell wall or involved in the formation of biofilms. The greatest effect was observed in the case of chitinases [67,68,69,70,71,72,73,74,75,76,77,78], among which there were both exo- and endochitinases. At the same time, their simultaneous presence in the enzyme complexes used to suppress the growth of fungi was the most successful [77,79].

As a number of studies have shown [79,97,98], such a combination of chitinases with different substrate ranges can be successfully supplemented by the action of other hydrolytic enzymes (proteases and glucanases) [87,88,96,97,98], which use molecules performing the role of structural elements of the fungal cell wall and membranes as substrates. However, it should be emphasized that not all chitinases known today [100] can be used as antifungal agents since the diverse structure of fungal polysaccharides is characterized by the presence of various glycoside residues of different lengths and often does not correspond to the preferences of those substrate specificities possessed by most of these enzymes. In addition, the levels of biosynthesis of these enzymes cannot meet the needs that arise even when studying their properties. In this regard, it becomes necessary to resort to obtaining their recombinant forms, where the most commonly used cells for this purpose are *E. coli* BL21 (DE3) [69,70,76,81,82].

It is important to note that yeast cells are usually difficult to destroy since their cell walls can form capsules or resistant spores. Using a complex of lysing enzymes such as Lyticase (a mixture of β-(1-3)-glucan-laminar-ipentaohydrolase, β-(1-3)-glucanase, protease, and mannanase), DNA can be extracted from yeast cells [98]. The activity of this complex induces the partial formation of spheroplasts; subsequently, the spheroplasts are lysed with the release of DNA.

Nucleases hydrolyzing the RNA and DNA of fungi attract particular attention among enzymes that have antifungal activity [93,94,99]. The use of several nucleases at once [96,97,98] or nuclease in combination with glucanase [99] leads to the fact that not only is the growth of fungal cells stopped, but membrane destruction (permeabilization and depolymerization) is observed. This reduces the membrane potential of mitochondria, leading to the degradation of target cell nucleic acids and the death of microbial cells. When giving priority to hydrolases when necessary to influence fungi, it can be emphasized that they generally have the potential for antifungal effects in a fairly wide range of pH values (3.0–11.5) and temperatures (up to 80 °C) (Table 1). At the same time, it should be noted that the activity of hydrolases strongly depends on the presence of various metals in the media of their functioning [69,70,73,74,78,85,88]. In such media, the most attractive options are those combinations of enzymes and metals that can significantly increase the effectiveness of the antifungal action of hydrolases. Among the metal ions, which in the largest number of studies have had a stimulating effect on the activity of hydrolases, Cu^2+^ [73,74] and Ca^2+^ [78,81,82,88] should be singled out, although their positive effect is not at all unambiguous, and in some cases, they had the opposite (inhibitory) effect on the hydrolytic activity of enzymes. At the same time, the positive results obtained during enzymatic reactions directed against fungi in environments in the presence of metals indicate the expediency of searching for possible combinations of metals and enzymes in the development of new antifungal formulations.

Oxidoreductases, in particular, peroxidases are standing in second place after hydrolases in popularity among enzymes used as potential antifungal agents [91,92]. These enzymes catalyze the oxidation of fungal molecules by reducing hydrogen peroxide (H_2_O_2_). The limitations in the use of these enzymes as antifungal agents are associated with a lower efficiency of their action in comparison with hydrolases and the need to introduce additional H_2_O_2_ into the medium with fungi.

### 3.2. Enzymes Hydrolyzing Fungal Proteins with Amyloid Characteristics

Special attention should be paid to the fact that yeast and mycelial fungi are able to form amyloids, which are unbranched fibrils consisting of monomers stacked on top of each other and stabilized by intermolecular β-layers. For example, monomers of hydrophobins of class I, small surface-active proteins produced by fungi, form amyloid fibrils that perform many functions [101]. It is known that the specific functions of hydrophobins synthesized by fungi can enhance their pathogenicity. Thus, *A. fumigatus* can cause invasive aspergillosis in patients with weakened immunity due to the amyloid-forming ability of hydrophobin RodA [102,103]. The formation of amyloid by hydrophobin MPG1 in *M. oryzae* contributes to rice pyriculariosis [104]. One of the most well-described examples of amyloid proteins in yeast cells is the Cdc19 protein from *S. cerevisiae*, which, in the absence of glucose, self-aggregates into an amyloid-like aggregate to avoid degradation under stressful conditions [105].

It is known that the yeast cells of *C. albicans*, often used in studies of antifungals, also contain proteins with amyloid characteristics. Thus, the proteins As1, As3, and As5 from the ALS-type adhesion family have the ability to self-aggregation. The presence of an amyloid sequence in the monomers of these proteins leads to the formation of hydrophobic nanodomains that promote the cell adhesion of *C. albicans* on biotic or abiotic surfaces and improve their ability to form biofilms [106,107]. It is assumed that Sap6, Rbt1, Page59, and Pga62 proteins, as well as adhesins, play a significant role in the appearance of *C. albicans* biofilms due to the presence of an amyloid-forming sequence in their structures [108,109,110,111].

Today, due to their ability to be transmitted from “mother” cells to “daughter” cells, yeast prions are classified as infectious, for example [URE3] and [PSI+], HET and HELLP in *S. cerevisiae*, *Podospora anserina*, and *Chaetomium globosum* cells, correspondently [112]. The presence of similar conditions for the formation of yeast prions and common molecular properties with pathogenic human amyloids has now led to the creation of models of neurodegenerative diseases based on yeast prions. The methods of their regulation are being investigated in order to develop new effective therapeutic agents and approaches to the treatment of diseases associated with prion proteins [113]. In this regard, the interest in enzymes capable of hydrolyzing amyloid aggregates formed by fungi is huge. This is due to the possible development of antifungals that reduce the level of biofilm formation and the potential use of enzyme-containing formulations for the treatment of neurodegenerative diseases in humans. Information about such proteases hydrolyzing amyloid proteins is presented in Table 4 [114,115,116,117,118,119,120,121,122,123,124,125,126,127,128,129].

Discussing the prospects for the possible use of enzymes hydrolyzing fungal amyloid proteins, it should be noted that so far there are a few such studies. The ability of several proteolytic enzymes, such as subtilisin, keratinases, and proteinase K, to degrade yeast prion aggregates of protein Sup35NM under various conditions was investigated [124,125,126,127]. It has been shown that hexameric AAA^+^-ATPase (Hsp104), which is a yeast chaperone, is involved in the fragmentation of large fungal amyloid fibrils. It is believed that the direct binding of Hsp104 to amyloid fibrils prevents the reproduction of yeast prions. Since Hsp104 is absent in the cells of multicellular animals, including mammals, the possibility of constructing variants of Hsp104 with the potential for use for the degradation of abnormal human proteins is being investigated [113].

Despite the limited number of studies in the field of enzymatic degradation of yeast prions, a number of proteolytic enzymes are known today that can degrade prion proteins and amyloids associated with human diseases, including subtilisin-like serine proteases TK-SP from hyperthermophilic archaeon *T. kodakarensis* [114], nattokinase from *Bacillus subtilis natto* [115], subtilisin 309 and protease from *B. lentus* [116,118], two prionzymes from *B. subtilis* and *B. lentus* [117,119], subtilisin-like protease MSK103 from *B. licheniforms* [120], enzyme E77 from *Streptomyces* sp. [121], subtilisin-homolog pernisine from the extremophile archaea *Aeropyrum pernix* [122], and serine protease from lichens [123].

Multiple metalloenzymes have been reported to have an important role in the degradation of Aβ [128,129], including two metal-activated keratinases, Ker1 and Ker2, from an actinomycete *Amycolatopsis* sp. MBRL 40; NEP—a zinc-dependent metalloprotease, cleaving various vasoactive peptides; and IDE—another zinc-dependent metallopeptidase, which could cleave insulin and amyloid Aβ. The ability to cleave amyloid precursor proteins has been confirmed in Zn-containing transmembrane metalloproteases [129]. At the same time, the influence of redox-active metals such as Cu and Fe (affecting the pathogenesis of Alzheimer’s disease) was established, which consists in increasing the biosynthesis of the metalloproteases under discussion. The influence of the same metals on the activity of these enzymes has not yet been investigated but is of great scientific and practical interest.

Despite the fact that there is still no effective enzymatic formulation for the cleavage of prion proteins, new proteolytic enzymes continue to be discovered and studied, the prionase activity of which still needs to be investigated [130,131,132]. In this review, we focus readers’ attention on such enzymes as a potential basis for the development of new antifungals, probably with some anti-neurodegenerative effect.

### 3.3. Enzymes Hydrolyzing Mycotoxins, Antibiotics, and QS Molecules (QSMs) of Fungi

To date, a significant amount of information has been accumulated about QS in the cells of various fungi and molecules that are produced by the fungi themselves in response to an increase in their concentration per unit volume. These QSMs are produced in order to trigger the processes of fungal cell transition to a state of stable intercellular communication, synchronization of the functions of multicellular populations, and biochemical changes in the cells themselves [133,134,135,136,137]. The ability of individual enzymes to catalyze the hydrolysis of fungal QSMs allows them to be attributed to the so-called Quorum Quenching enzymes (QQE). Gluconolactonase- [138]) and hexahistidine-containing organophosphorus hydrolase (His_6_-OPH) [139,140] esterases [141,142]) have been identified as such enzymes acting against fungi today (Table 5).

Discussing the potential of these enzymes as candidates for inclusion in combined antifungals with metal-containing compounds, it can be noted that for His_6_-OPH, such possibilities have already been demonstrated and proved promising, while Ta NPs [146,147] appeared to be the most effective option for such a combination. However, so far, such combined antimicrobials have been investigated only against bacterial cells [148], and their effectiveness against fungal cells has yet to be confirmed.

Interesting use cases for combining with metal-containing compounds are enzymes that carry out the destruction of mycotoxins synthesized by fungi in the CFR state. At the same time, it should be noted that, as in the case of CSM hydrolysis, among the enzymes that carry out the destruction of various mycotoxins (zearalenone, patulin, deoxynivalenol, ochratoxin), there are all the same enzymes that are listed in Table 5, namely lactonases, esterases, lipases [22], and His_6_-OPH [65,149]. In this regard, with their involvement in combined antifungal formulations, a very interesting option may turn out to provide a multi-targeted action due to the promiscuous activities of these enzymes.

Continuing to analyze possible variants of enzymes that can be considered as candidates for creating combined variants with metal NPs, it is undoubtedly necessary to pay attention to enzymes that are able to catalyze the hydrolysis of antibiotics synthesized by fungi among other secondary metabolites in their QS state. Here, the undisputed leaders are β-lactamases, known to everyone due to studies of bacterial antibiotic resistance to natural and semi-synthetic penicillins and cephalosporins [150].

It is interesting to note that QQE including His_6_-OPH are close “relatives” for metallo-β-lactamases [151]. Moreover, the structural analogy revealed between phosphotriesterase (of the same His_6_-OPH) and some nucleases indicate that all these enzymes can catalyze to one degree or another similar reactions with a certain preference for individual substrates. Since these enzymes have been mentioned here more than once in connection with their various targets of action in fungal cells (Table 3, Table 4 and Table 5), their use in research on the development of new antifungals may be not only new but also promising. Surprisingly, an active search for data on the use of metallo-β-lactamases in the content of any antifungals to give them a number of catalytic activities, as discussed above, did not reveal any.

It should be noted here that many of mentioned enzymes contain different transition metals, particularly Zn(II), Mn(II), and Fe(II)/Fe(III) in their active sites [151], which can be positively taken into account when creating combinations with metal-containing compounds since there are fungi sensitive to these metals (Table 1 and Table 2). In addition, the combination of these enzymes with metal-containing compounds that are not embedded in the active site of enzymes but can exhibit significant antimicrobial activity at low MIC values [146,147] looks interesting and promising.

## 4. Combination of Antifungal Enzymes and Metal-Nanoparticles

It is known currently that many sources and types of enzymes can be used to prepare antifungal formulations with metal NPs: bacterial keratinase [152] and chitinase [153]; archaeal protease and lipase [154]; fungal β-1,3-glucanase, N-acetylglucosaminidase, chitinase, and acid protease [155,156], etc. Such formulations can possess secondary antioxidant [152,153] and/or specific inhibitory activity [152]. The additional antibacterial action mode of these combinations is widely present [152,154,156,157] (Table 6 [42,152,153,154,155,156,157,158]).

“Green synthesized” metal NPs are of great interest for the production of enzyme formulations [152,154,155,156]. β-1,3-glucanase(s) and, to a lesser extent, N-acetylglucosaminidase(s) are prevalently adsorbed by Ag NPs as compared to chitinase(s) and acid protease(s) [155]. Altogether, these enzymes on Ag NPs not only inhibit mycelium growth but also prevent the formation of sclerotia thereby leading to lifecycle arrest.

Interestingly, the “un-capping” of Ag NPs (i.e., desorption of enzymes) leads to a detectable increase of their size and is likely to be a result of their aggregation [156]. At the same time, the negative net charge of “uncapped” Ag NPs argues for the substitution of enzymes for sodium dodecylsulfate used as a solubilizer. This can contribute to the increased toxicity of such “un-capped” NPs towards non-target organisms and cell lines [156]. Surprisingly, “un-capped” Ag NPs are ineffective in a mycelium growth test and only decrease the number of sclerotia by twofold as compared to the control experiment without any effector.

Similar to germination, the formation of sclerotia is known to be regulated by multiple genes though there are a lot of gaps in our knowledge about this process [159]. As a result, the biochemical composition of the cell wall changes dramatically; for example, the most abundant components of *Sclerotium rolfsii* hyphae—polysaccharides and lipids—shift by 1.5–2 times (down and up, respectively), while unhydrolyzable compounds (so-called ‘melanin-like pigments’) increase numerously and become the second prevalent subclass (after polysaccharides). The last ones have been shown to propagate resistance of sclerotia towards environmental factors and, for example, to slaughter via the hydrolytic action of extracellular glucanases and chitinases [160]. Moreover, the leakless thick rind can be formed from such melanized cells on the sclerotia surface [161], further limiting enzymatic hydrolysis and antifungal penetration. Thus, polyphenol-degrading activity may be useful in addition to antifungal formulation. Another rational functionality in such formulation(s) to treat sclerotia appears to be the antioxidant activity discussed previously since ROS also affects sclerotial development somehow [159].

During field trials of chitinase-based formulation against filamentous fungi [153], it was found to be slightly less effective than the same formulation with a live biocontrol agent (*Streptomyces cellulosae*). This may be a consequence of differing profiles of protective gene modulation in the plant by these formulations.

As was determined for peptide melittin, a slow release of active compound from the Zn-MOF matrix occurs and the maximal amount (60%) is released at pH 6 during 24 h [158]. The antifungal activity of melittin is naturally decreased threefold during its encapsulation within Zn-MOF at 30 wt.% loading. However, lactoferrin added to such a formulation improves it almost twofold. Altogether, yeast adhesion to the surface during biofilm formation and (pseudo)hyphal transformation are inhibited.

Melittin is known to disturb membranes of different (micro)organisms, activate several transmembrane receptors, depolarize membranes, etc. Some of these effects are also manifested in the composite formulation [158]. Moreover, lactoferrin being a transporter of iron ions and having other possible activities [162] greatly improves the antifungal activity of melittin, especially towards pre-formed biofilms [158]. The synergic action of lactoferrin and melittin can also be detected using an animal infection model in vivo. Lactoferrin can bind to the fungal cell surface itself and affect biofilm formation and yeast-to-hyphal transition in combination with conventional drugs [163]. Thus, lactoferrin and melittin may interact with multiple and differing targets on the yeast cell wall and within the cell while amplifying the antifungal activity of each other.

Some toxicity was shown for Ag NPs toward the lung fibroblasts of Chinese hamsters, the embryo fibroblasts of albino Swiss mice, human aneuploid immortal keratinocytes, and the roots of onions [155,156]. Moreover, such formulations affect the soil microbial (bacteria and fungus) community in situ after single exposure for at least 360 days [156]. It is interesting that the toxicity of such polypeptide as melittin toward the macrophage cell line from a mouse tumor is greatly decreased within Zn-MOF formulation [158]. However, since doses of melittin in free and encapsulated form were discrepant then, total removal of toxicity cannot be concluded now.

## 5. Conclusions

After analyzing the approaches to the development and combination of antifungals that were discussed 10 years ago [164] and those that are currently discussed [165], it can be noted that there is not much difference between them, and there have been no discussions of solutions based on the use of metal-containing compounds and enzymes and even more so on their possible combinations. At the same time, the expediency and possible effectiveness of the combination of substances that enable the use of different mechanisms for suppressing the growth and metabolic activity of fungi have long been beyond doubt. It remains only to solve the problem of choosing partners for the most effective and safe combination of antifungal agents for humans. The emergence of new knowledge about possible targets for exposure to fungi and the analysis of the palette of known antifungal agents can form fresh ideas about possible useful combinations. Of course, those variants are interesting in terms of not just the inhibitors of biochemical processes that are used, which, as a rule, react with their targets in a one-to-one ratio and require specific binding, but namely because biocatalysts are attractive for the processes of irreversible degradation of key fungal biomolecules that repeatedly enter into decisive catalytic acts.

## Figures and Tables

**Table 1:** Antifungals based on metal nanoparticles (NPs), metal–organic frameworks (MOFs), and their composites *.

Antifungal Agent [Reference]	Target of Action	Antifungal Activity	Efficiency of Antifungal Action
ZrO_2_-Ag_2_O (14–42 nm) [23]	*Candida albicans*, *C. dubliniensis*, *C. glabrata*, *C. tropicalis*	The growth rate inhibition	89–97% inhibition
WS_2_/ZnO nano-hybrids [24]	*C. albicans*	Inhibition of biofilm formation	91% inhibition
CuO@C (36–123 nm) [25]	*Alternaria alternata*, *Fusarium oxysporum*, *Penicillium digitatum*, *Rhizopus oryzae*	Inhibition of the hydrolytic activity of fungal enzymes used by them for their own metabolism	Inhibition (100 μg/mL) of cellulases and amylases secreted by fungi: 38% and 42% for *A. alternata*, 39% and 45% for *F. oxysporum*, 24% and 67% for *P. digitatum*, and 20% and 24%for *R. oryzae*, respectively
ZnO NPs [26]	*C. albicans*, *Aspergillus niger*	Inhibition of growth	Large enough zone of growth absence (8-9 mm)
ZnO NPs (20–45 nm) [27]	*Erythricium salmonicolor*	Notable thinning of the hyphae and cell walls, liquefaction of the cytoplasmic content with decrease in presence of a number of vacuoles	Significant inhibition (9–12 mmol/L) of cell growth
ZnO–TiO_2_ NPs (8–33 nm) [28]	*A. flavus*	High level of ROS production and oxidative stress induction. Treated objects have a lower count of spores and damaged tubular filaments and noticeably thinner hyphae compared to the untreated fungi	Fungicidal inhibition (150 μg/mL) zone is 100 %
ZnO NPs (40–50 nm) [29]	*C. albicans*	High level of ROS production	MIC = 32–64 μg/mL MFC = 128–512 mg/mL
Fe_2_O_3_ NPs (10–30 nm) [30]	*Trichothecium roseum*, *Cladosporium herbarum*, *P. chrysogenum*, *A. alternata*, *A. niger*	Inhibition of spore germination	MIC = 0.063–0.016 mg/mL
Fe_3_O_4_ NPs (70 nm) [31]	*C. albicans*	Inhibition of cell growth and biofilm formation	MIC = 100 ppm MFC = 200 ppm
Cu-BTC (10–20 µm) [32]	*C. albicans*, *A. niger*, *A. oryzae*, *F. oxysporum*	ROS producing, the damage of the cell membrane	Inhibition of *C. albicans* colonies is 96% by 300 ppm and up to 100% by 500 ppm. Inhibition growth of *F. oxysporum* and *A. oryzae* is 30% with 500 ppm. No significant effect on the *A. niger* growth.
HKUST-1 or HKUST-1 NPs (doped with NPs of Cu(I)) (49–51 nm) [33]	*A. niger*, *F. solani*, *P. chrysogenum*	Appearance of Cu^+2^ inhibiting of cell growth	100% growth inhibition of *F. solani* by 750–1000 ppm and *P. chrysogenum* by 1000 ppm; for *A. niger*—no inhibition
[Cu_2_(Glt)_2_(LIGAND)] (H_2_O) [34]	*C. albicans*, *A. niger* spores	The apoptosis-like fungal cell death, ROS production	50–70% death of *C. albicans* and 50–80% germination inhibition of *A. niger* at 2 mg/mL of the MOFs
MIL-53(Fe) and Ag@MIL-53(Fe) composite [35]	*A. flavus*	Inhibition of cell growth	MIC = 40 μg/mL for the MIL-53(Fe); MIC = 15 μg/mL for the Ag@MIL-53(Fe)
MOF on the basis of Ce and 4,4′,4″-nitrilotribenzoic acid [11]	*A. flavus*, *A. niger*, *Aspergillus terreus*, *C. albicans*, *Rhodotorula glutinis*	Enzyme-like activity: catalase, superoxide dismutase, and peroxidase	Inhibition efficiency of 93.3–99.3% based on the colony-forming unit method
TiO_2_ co-doped with nitrogen and fluorine (200–300 nm) [12]	*F. oxysporum*	Peroxidase-like activity, production of ROS under light irradiation	100% inhibition of fungal growth
Fe_3_O_4_@MoS_2_-Ag (~428.9 nm) [36]	*C. albicans*	Peroxidase-like activity	80% damage of cell membranes
CoZnO/MoS_2_ nanocomposite [37]	*A. flavus*	Peroxidase-like activity under light irradiation	MIC = 1.8 mg/mL

* BTC—1,3,5-benzenetricarboxylate; Glt—glutarate; HKUST-1—type of MOFs composed of [Cu_3_(BTC)_2_(H_2_O)_3_]_n_; MFC—minimum fungicidal concentrations; MIC—minimum inhibitory concentration; MIL-53(Fe)—type of MOFs composed of [Fe_4_(OH)(1,4-benzenedicarboxylate)_4_]; LIGAND—1,2-bis(4-pyridyl)ethane, 1,2-bis(4-pyridyl)ethylene, or 1,3-bis(4-pyridyl) propane.

**Table 2:** Metal-containing antifungal formulations synthesized via green route.

Antifungal Formulation [References]	Target Fungi	* MFC, mg/L	Comment
Green synthesized Au NPs in *Spirulina maxima* [10]	*C. albicans*	0.064 (μDT)	Damage of cell wall
Green synthesized Ag NPs in *Desmodesmus* sp. [47]	*C. parapsilosis*	n/a	Antifungal agent
Green synthesized Ag NPs (30 nm) [48]	*C. albicans*	n/a (DDT)	Antibacterial agent
Bio-synthesized Te nanorods (ca. 50 × 500 nm) [49]	*F. oxysporum* *A. alternata*	60 (SGT) 40 (SGT)	Germination inhibitor
Green synthesized Ag NPs (30 nm) [50]	*C. albicans* *C. glabrata* *C. parapsilosis*	1.6–6.3 (μDT) 3.1 (μDT) 12.5 (μDT)	Antibiofilm agent, yeast-to-hyphal inhibitor
Green synthesized Fe_3_O_4_ NPs (0.5–2 μm) [51]	*C. albicans*	6.3 (μDT)	Antibiofilm agent, yeast-to-hyphal inhibitor
Green synthesized Ti/Ag NPs (20–120 nm) [52]	*A. niger*, *A. flavus*, *F. solani*	>100 (MGT)	Antibacterial agent
ZnO microparticles (2–5 μm) in film of soybean proteins with cinnamaldehyde [53]	*A. niger*	n/a (DDT)	Coating for surfaces, field trial
ZnO NPs (20–40 nm) in zein/gelatin nanofibers with (poly)phenolic acids [54]	*Botrytis cinerea*	n/a (MGT)	Coating for surfaces, field trial
CuO NPs in Ca-alginate nanogel coated by poly-ε-lysine (60 nm) [55]	*A. alternata*, *B. cinerea*, *Phytophthora capsica*, *Thanatephorus cucumeris*, *F. graminearum*	>1000 (MGT)	Germination inhibitor, field trial

* MFC—minimum fungicidal concentration; n/a—not available; DDT—disk diffusion test; μDT—microdilution test; MGT—mycelium growth test; SGT—spore germination test.

**Table 3:** Enzymes from various sources with antifungal activity.

Enzyme, Its Molecular Weight and Origin [Reference]	Object of Action	Conditions of Action	Target of Action
**Chitinases**			
Chitinase (35 kDa) from seeds of naked oat *Avena chinensis* [67]	*Panus conchatus*, *Trichoderma reesei*	pH 7.0; 30-50 °C	Hydrolysis of β-1, 4-glycosidic linkages in chitin (an insoluble linear homopolymer of β-1,4-linked *N*-acetyl-glucosamine residues
Chitinase (33 kDa) from *Lactobacillus coryniformis* 3N11[68]	*Alternaria alternata*	pH 5.0–7.0; 50–80 °C	Inhibition of fungal cell growth detected by the method of disks on agar-containing medium
Acidic chitinase (52.8 kDa) from *Paenibacillus xylanexedens* Z2–4 was produced in *Escherichia coli* BL21 (DE3) cells [69]	*Alternaria alstroemeriae*, *Botrytis cinerea*, *Rhizoctonia solani*, *Sclerotinia sclerotorum*, *Valsa mali*	pH 4.0–13.0; 50–65 °C; pH_optimum_ 4.5; Inhibition of the enzyme was detected by the metals: 22-24% by Cu^2+^ and Co^2+^; 15% by Cr^3+^ and Mn^2+^; 17–18% by Sr^2+^, Ni^2+^, Fe^2+^.	The highest specific activity has towards colloidal chitin, followed by ethylene glycol chitin and ball milled chitin. It inhibits the hyphal extension.
Chitinase (30 kDa) from *Drosera rotundifolia* was produced in *Escherichia coli* BL21-CodonPlus (DE3)-RIPL [70]	*Fusarium poae*, *Trichoderma viride*, *Alternaria solani*	pH 5.0–7.0; 30–45 °C; Inhibition of the enzyme was detected by the metals: 17% by Fe^2+^, 40% by Pb^2+^, 48% by Cu^2+^ and 58% by Cd^2+^	40-52.6% decrease of fungal growth, whereas the stimulation (up to 50%) of growth of *R. solani* sp.
Chitinase (64.1 kDa) from *Bacillus amyloliquefaciens* [71,72]	*Botryosphaeria dothidea*, *B. cinerea*, *F. graminearum,* *Rhizoctonia cerealis*, *S. sclerotiorum*, *Ustilaginoidea virens*	pH 8.0; 37 °C	60–68% decrease of fungal growth, degeneration of hyphae morphology
Chitinase (77.9 kDa) from a rare actinomycete *Saccharothrix yanglingensis* Hhs.015 (isolated from the roots of cucumber) [73]	*V. mali*	pH 6.0; 30–45 °C; Inhibition of the enzyme was detected by the metals: 80% by Zn^2+^, 65-70% by Ca^2+^, Mn^2+^, Fe^2+^. Ions Cu^2+^, Cr^3+^ and Mg^2+^ significantly promoted chitinase activity (by 187.3%, 167.5% and 111.9%, respectively).	Multiple deformations of fungal hyphae.
Chitinase (45 kDa) from *Streptomyces luridiscabiei* U05 (isolated from wheat rhizosphere) [74]	*A. alternata*, *F. oxysporum*, *F. solani*, *F. culmorum*, *B. cinerea*, *Penicillium verrucosum*	pH 6.0–8.0; 35–40 °C; 98% inhibition of the enzyme was detected in presence of Hg^2+^ and Pb^2+^. The chitinase activity was stimulated by Ca^2+^ (120%) and Mg^2+^ (140%) ions.	Inhibition of fungal growth due to the demonstration of both endo- and exochitinase activity by the enzyme.
Chitinase (94.2 kDa) from *Salinivibrio* sp. BAO-1801 [75]	*A. niger*, *F. oxysporum*, *R. solani*	pH 6.0–8.0; 40–55 °C	100% inhibitory effect on spore germination of fungal cells
Endochitinase (52.9 kDa) from *Corallococcus* sp. EGB was produced in *Escherichia coli* BL21 (DE3) [76]	*Magnaporthe oryzae*	pH 5.0–8.0; 30–55 °C	Inhibition of conidia germination and appressorium formation due to the hydrolysis of chitin into *N*-acetylated chitohexaose
Endo- and exochitinases (34, 41 and 48 kDa) from *Serratia marcescens* PRNK-1 isolated from cockroaches *Periplaneta americana* [77]	*R. solani*, *F. oxysporum*	pH 4.5–7.0; 40–60 °C; pH_optimum_ 5.5, t_optimum_ 55 °C	Strong inhibition of fungal hyphae growth
Chitinase (46 kDa) from *Trichoderma harzianum* GIM 3.442 [78]	*B. cinerea*	pH 5.0–8.0; 40–55 °C; pH_optimum_ 6.0, t_optimum_ 45 °C 49.4% and 66.6% inhibition of the enzyme was detected in presence of Zn^2+^ and Cu^2+^ respectively. The chitinase activity was stimulated by Ca^2+^ (115.2%) and Sr^2+^ (112.6%) ions.	Up to 80% inhibition of fungal growth
Complex of chitinases (25, 37 and 110 kDa) from *Aeromonas* sp. [79]	*F. solani*, *A. alternate*, *B. cinerea*, *Penicillium* sp.	pH 5.0–8.0; 30-50 °C	Inhibition of fungal growth
Endochitosanasa (50.7 kDa) from *Aquabacterium* sp. [80]	*M. oryzae*, *F. oxysporum*	pH 5.0; 40 °C	The enzyme inhibits appressorium formation of *M. oryzae and* hydrolyzes 95%-deacetylated chitosan with accumulation of chitooligosaccharides inhibiting the growth of fungal cells of *M. oryzae* and *F. oxysporum*.
Chitinase (30 kDa or 48 kDa) from *Streptomyces sampsonii* with bifunctional activity was produced in *Escherichia coli* BL21 (DE3) [81,82]	*Cylindrocladium scoparium*, *Cryphonectria parasitica*, *Neofusicoccum parvum*, *F. oxysporum*	pH 3.0–11.5; 30–60 °C pH_optimum_ 6.0, t_optimum_ 55 °C Enzymatic activity was stimulated by Ca^2+^ (132%), Mg^2+^ and Mn^2+^ slightly (9%) decrease the activity, whereas Ag^+^ and Cr^3+^ notably inhibited enzymatic activity (80% and 42% respectively).	The enzyme possessed the dual enzymatic activity of chitinase and lysozyme and its action results in complete destruction of the mycelial morphology.
**Glucanases**			
Endo-1,4-galactosaminidase from *Aspergillus fumigatus* was produced in *E. coli* BL21 (DE3) [83]	*A. fumigatus*	pH 6.0-7.0; 28 °C	Enzyme catalyzes the hydrolysis of exopolysaccharide galactosaminogalactan being integral component of *A. fumigatus* matrix
Endo-a-1,4-N-acetyl-d-galactosaminidase (produced in *E. coli*) and Endo-a-1,4-d-galactosaminidase (produced in *Pichia pastoris*) [84]	*A. fumigatus*	pH 7.4; 37 °C	The enzymes catalyze destruction of adhesive exopolysaccharides in biofilms formed by fungi.
Endo-β-1,3-glucanase (46.6 kDa) from *M. oryzae* was produced in *E. coli* BL21 (DE3) [85]	*M. oryzae*, *U. maydis*	pH 5.0–8.0; 20–45 °C Relative activity was following in presence of K^+^ (39%), Ba^2+^ (46.2%), Ca^2+^ (47.1%), Co^2+^ (22.2%), Cr^2+^ (55.1%), Cu^2+^ (30%), Mg^2+^ (31.6%), Mn^2+^ (23.1%), Ni^2+^ (20.7%), Zn^2+^ (1.9%), Fe^3+^ (60%) and Fe^2+^ (103.1%)	The enzyme inhibits formation of germ tubes and appressoria.
β-(1-3)-glucanase (32 kDa) from *Bacillus halotolerans* was produced in *E. coli* BL21 [86]	*V. dahliae*	pH 7.0; 28 °C	Strong inhibition of spore germination and mycelial growth of the fungal cells.
**Proteases**			
Serine protease (87.16 kDa) from *B. licheniformis* TG116 [87]	*P. capsica*, *R. solani*, *F. graminearum*, *F. oxysporum*, *Botrytis cinerea*, *Cescospora capsici*	pH 7.3; 30 °C	The most notable inhibition of fungal growth was revealed in case of *C. capsici.*
Aspartic protease P6281 (38 kDa) from *T. harzianum was produced in Pichia pastoris* cells [88]	*B. cinerea*, *Mucor circinelloides*, *A. fumigatus*, *A. flavus*, *R. solani*, *C. albicans*	pH 2.5–4.0; 30–45 °C; 49.4% and 66.6% inhibition of the enzyme was detected in presence of Zn^2+^ and Cu^2+^ respectively. The aspartic protease activity was stimulated by Mn^2+^ (140.1%) and Cu^2+^ (151.2%) ions. Ca^2+^, Mg^2+^ and Ni^2+^ slightly (7–10%) increase the activity, whereas Fe^2+^ and Zn^2+^ ions decrease activity of the enzyme.	Inhibition of spore germination and growth of fungal cells: *57.3% B. cinerea*, 30.9% 26.1% *M. circinelloides*, 27.2% *A. fumigatus*, 34.8% *A. flavus*, *R. solani*, *C. albicans*
**Lysozyme**			
Lysozyme with fluconazole in shellac NPs [89]	*Biofilm of C. albicans*	pH 5.5	Biofilm clearing effect was observed.
Lysozyme in NPs of chitosane [90]	*Aspergillus parasiticus*	28 °C	The decrease in fungal cell viability, 100%- inhibitory effect on the germination of spores was confirmed.
**Peroxidases**			
Peroxidase (58 kDa) from cowpea (*Vigna unguiculata*) roots [91]	*Colletotrichum gloeosporioides,* *F. oxysporum*	pH 4–7; 37–75 °C	Enzyme catalyzes redox reactions and inhibits the conidia germination of fungal cells by altering the permeabilization of membranes.
Peroxiredoxin (38 kDa) from *Enterobacter* sp. was produced in *E. coli* DH5α [92]	*Verticillium dahlia*, *F. solani*	pH 4–7; 37–75 °C	Peroxidase inhibits the growth of fungi.
**Nucleases**			
RNase 3 from eosinophils and the skin-derived RNase 7 were produced in *E. coli* BL21 (DE3) [93,94]	*C. albicans*	pH 5.0–7.2; 20–37 °C	RNases demonstrated dual mechanism of action: an overall yeast membrane-destabilization (permeabilization and depolymerization) and degradation of target cellular RNA.
Bovine pancreas RNase A1, human recombinant ribonucleases A2, A5 and A8 [95]	*C. albicans*, *C. glabrata*	pH 5.0–7.2; 30–37 °C	Action of RNase A1 was the most pronounced, it completely killed *Candida* cells by lowering the mitochondrial membrane potential but did not damage the cell membrane.
**Enzymatic comlexes**			
Chitinase and β-1,4-endoglucanase co-synthesized by *Paenibacillus elgii* PB1[96]	*A. niger*, *C. albicans*, *Trichophyton rubrum*, *Microsporum gypseum*, *Saccharomyces cerevisiae*	pH 5.0; 30 °C Urea had significant negative effect on β-1,4-endoglucanase, Zn^2+^ positively affected both enzymatic activities.	Inhibition of fungal growth: 88% *A. niger,* 92% *T. rubrum*, 52% *M. gypseum*, 55% *C. albicans*, 71% *S. cerevisiae*
Enzymatic complex from *Penicillilum verruculosum* containing chitinase (43 kDa, gene from *Myceliophtora thermophyla*), cellobiohydrolase (66 kDa), endoglucanase (39 kDa) and xylanase (32 kDa) [97]	*Fusarium culmorum*; *F. sambucinum*; *F. graminearum*; *Stagonospora nodorum*; *S. tritici*; *A. flavus*	pH 4.5–6.2; 52–65 °C	The enzymatic complex catalyzed hydrolysis of fungal mycelium.
Lyticase (enzymatic complex with activity of β-(1-3)-glucan laminaripentaohydrolase, β-(1-3)-glucanase, protease and mannanase) from *Arthrobacter luteus* [98]	*C. albicans*	pH 7.3; 25–37 °C	Lyticase provides disruption of yeast cell walls and spores with formation of spheroplasts and further release of DNA from them.
Cellobiose dehydrogenase (CDH) and deoxyribonuclease I (DNase) co-immobilized on positively charged chitosan nanoparticles [99]	Polymicrobial biofilms of *C. albicans* and *Staphylococcus aureus*	pH 7.5; 37 °C	The action of two enzymes provides a violation of biofilm formation due to the degradation of eDNA, a decrease in the thickness of the biofilm and the death of microbial cells.

**Table 4:** Different proteases applied for prion degradation.

Enzyme; Origin; Reference	Protease Type	Prion/Amyloid
Subtilisin homolog Tk-SP from *Thermococcus kodakarensis* [114]	Serine protease	abnormal human prion protein
Nattokinase from *Bacillus subtilis natto* [115]		amyloid β fibrils/recombinant human prion protein
Subtilisin 309 from *Bacillus lentus* [116]		mouse-adapted scrapie prion protein
Prionzyme from *Bacillus subtilis* [117]		hamster prion protein
Properase from *Bacillus lentus* [118]		mouse-adapted prion protein
MC3 (Prionzyme) from *Bacillus lentus* [119]		301V prion
MSK103 from *Bacillus licheniforms* [120]		hamster-adapted scrapie prion protein
E77 from *Streptomyces* sp. [121]		
Pernisine from *Aeropyrum pernix* [122]		abnormal human prion protein
Protease from lichens (*Parmelia sulcata*, *Cladonia rangiferina* and *Lobaria pulmonaria*) [123]		hamster prion protein
Keratinase from *Bacillus licheniformis* [124]		yeast prion protein, Sup35NM
Proteinase K [125]		
Keratinase rKP2 from *Pseudomonas aeruginosa* KS-1 [126]		
Keratinase from *Bacillus pumilus* KS12 [127]		
Keratinases, Ker1, and Ker2, from *Amycolatopsis* sp. MBRL 40 [128]	Metal-activated serine protease	amyloid β fibrills
Neprilysin [129]	Zn-dependent metalloprotease	amyloid β fibrills
Insulin-degrading enzyme [129]		
A disintegrin and metalloproteinase (ADAM10) [129]		

**Table 5:** Enzymes hydrolyzing the QSMs of various fungal cells.

QQE [Reference]	Objects of Action	Target QSMs
Gluconolactonase [138]	*A. niger*	Lactone-containing QSMs
His_6_-OPH [139,140]	*Trichosporon beigelii*, *Candida* sp., *S. cerevisiae*, *Pachysolen tannophilus*, *Kluyveromyces marxianus*	Lactone-containing QSMs
Esterases [141,142]	*Mucor mucedo*, *Blakeslea trispor* *Phycomyces blakesleeanus*	Trisporic acids
Lipases [143,144,145]	*Malassezia* sp., *Microsporum canis* *Leishmania amazonensi*	Lipids with long carbon chain fatty (oleic, linoleic, and linolenic) acids

**Table 6:** Combined application of enzymes possessing antifungal activity with metal NPs.

Antifungal formulation [Reference]	Target Fungi	MFC *, mg/L	Comment, [Reference]
Glucose oxidase with NPs of Fe_3_O_4_ [42]	*C. albicans*	1 mg/mL	Antimicrobial activity
Keratinase on green synthesized Ag NPs (5–25 nm) [152]	*C. albicans*	n/a (DDT)	Antibacterial agent
Chitinase on talc (0.2–3 μm) combined with chitin [153]	*Sclerotium rolfsii*	n/a	Passed field trial
Protease and lipase on green synthesized Ag NPs (10–45 nm) [154]	*C. albicans*	n/a (DDT)	Antibacterial agent
β-1,3-glucanase, chitinase, N-acetylglucosaminidase, and acid protease on green synthesized Ag NPs (20–200 nm) [155]	*Sclerotinia sclerotiorum*	n/a (MGT)	Variable sorption capacity of Ag NPs for different enzymes
β-1,3-glucanase, chitinase, N-acetylglucosaminidase and acid protease on green synthesized Ag NPs (20–200 nm) [156]	*S. sclerotiorum* *Beauveria bassiana*	n/a (MGT)	Antibacterial agent
Glucose oxidase conjugated with polyglutaraldehyde–β-alanin and covered by Ag shell [157]	*C. albicans* *Microsporum canis* *T. rubrum*	n/a (DDT)	Antibacterial agent, cytochrome inhibition
Lactoferrin and melittin in Zn–MOF (0.5 μm) [158]	*C.albicans*	>100 (μDT)	Antibiofilm agent, yeast-to-hyphal inhibitor used in vivo

* MFC—minimum fungicidal concentration; n/a—not available; DDT—disk diffusion test; MGT—mycelium growth test; μDT—microdilutin test.

## Data Availability

Not applicable.
